# Poly (d, l-lactide)/polyvinyl alcohol-based injectable microspheres with inflammation alleviation and cartilage regeneration enhancement for treatment of temporomandibular joints osteoarthritis

**DOI:** 10.1093/rb/rbag027

**Published:** 2026-03-02

**Authors:** Gu Cheng, Tao Huang, Lei Xu, Zongli Li, Xin Nie, Zubing Li, Tong Cao, Dongdong Xu, Yuting Yang, Zhi Li

**Affiliations:** School and Hospital of Stomatology, Wenzhou Medical University, Wenzhou 325000, Zhejiang, China; State Key Laboratory of Oral & Maxillofacial Reconstruction and Regeneration, Key Laboratory of Oral Biomedicine Ministry of Education, Hubei Key Laboratory of Stomatology, School & Hospital of Stomatology, Wuhan University, Wuhan 430079, China; Oujiang Laboratory (Zhejiang Lab for Regenerative Medicine, Vision and Brain Health), Wenzhou Medical University, Wenzhou 325000, Zhejiang, China; School and Hospital of Stomatology, Wenzhou Medical University, Wenzhou 325000, Zhejiang, China; Oujiang Laboratory (Zhejiang Lab for Regenerative Medicine, Vision and Brain Health), Wenzhou Medical University, Wenzhou 325000, Zhejiang, China; School and Hospital of Stomatology, Wenzhou Medical University, Wenzhou 325000, Zhejiang, China; School and Hospital of Stomatology, Wenzhou Medical University, Wenzhou 325000, Zhejiang, China; State Key Laboratory of Oral & Maxillofacial Reconstruction and Regeneration, Key Laboratory of Oral Biomedicine Ministry of Education, Hubei Key Laboratory of Stomatology, School & Hospital of Stomatology, Wuhan University, Wuhan 430079, China; School and Hospital of Stomatology, Wenzhou Medical University, Wenzhou 325000, Zhejiang, China; Oujiang Laboratory (Zhejiang Lab for Regenerative Medicine, Vision and Brain Health), Wenzhou Medical University, Wenzhou 325000, Zhejiang, China; School and Hospital of Stomatology, Wenzhou Medical University, Wenzhou 325000, Zhejiang, China; State Key Laboratory of Oral & Maxillofacial Reconstruction and Regeneration, Key Laboratory of Oral Biomedicine Ministry of Education, Hubei Key Laboratory of Stomatology, School & Hospital of Stomatology, Wuhan University, Wuhan 430079, China; Department of Stomatology, Shanghai East Hospital, School of Medicine, Tongji University, Shanghai 200120, China; State Key Laboratory of Oral & Maxillofacial Reconstruction and Regeneration, Key Laboratory of Oral Biomedicine Ministry of Education, Hubei Key Laboratory of Stomatology, School & Hospital of Stomatology, Wuhan University, Wuhan 430079, China

**Keywords:** osteoarthritis, drug releasing, inflammation, chondrogenesis, microspheres

## Abstract

Osteoarthritis (OA) is a prevalent joint disease characterizedby chronic, progressive inflammation and cartilage degeneration, for which current treatments remain limited. In this study, we propose a dual-drug delivery strategy that simultaneously suppresses inflammation and rejuvenates impaired cartilage by incorporating kartogenin (KGN) and methylprednisolone hemisuccinate (MPHS) into a single microsphere system with sequential release in the local microenvironment. To achieve coordinated dual-drug release, KGN and MPHS were loaded into the inner core and outer layer of the microspheres, respectively. Both KGN and MPHS exhibited sustained release profiles; however, MPHS showed a shorter burst-release phase than KGN due to the protective effect of the outer layer. The release of MPHS effectively suppressed interleukin-1β (IL-1β)-induced inflammation in bone marrow stromal cells (BMSCs) pellets, thereby enhancing KGN-mediated chondrogenic differentiation of BMSCs *in vitro*. In parallel, sustained delivery of KGN also led to the recruitment of BMSCs and subsequent chondrogenesis, ultimately leading to cartilage rejuvenation. Importantly, the sequential release of KGN and MPHS from the dual-drug microspheres synergistically enhanced *in vitro* chondrogenic differentiation of BMSCs, resulting in concomitant inflammation alleviation and cartilage repair. Collectively, these findings demonstrate that the KGN/MPHS-incorporated microspheres possess dual chondrogenic and anti-inflammatory functions and represent a promising therapeutic strategy for OA treatment.

## Graphical Abstract

**Graphical Abstract:**
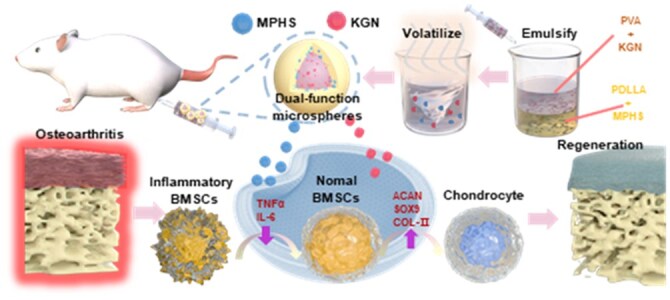

## Introduction

Articular cartilage, with its sophisticated architecture and composition, is capable of withstanding complex mechanical stresses, including shear, compression and friction [1]. Despite these properties, articular cartilage is frequently damaged by traumatic injury and age-related degeneration. Once injured, cartilage tissue exhibits a limited capacity for self-repair, primarily owing to its avascular nature and the restricted migratory potential of resident cells toward the damaged site [2]. Furthermore, cartilage injury often initiates an inflammatory response in the articular joint, characterized by elevated expression of synovial cytokines, which further accelerates tissue degradation. Ultimately, these biochemical and mechanical alterations ultimately contribute to the development of osteoarthritis (OA), a chronic and progressive degenerative joint disease affecting a large population worldwide [3].

OA is generally associated with markedly reduced joint lubrication, leading to synovial inflammation, progressive or irreversible cartilage degeneration and subchondral sclerosis [4, 5]. Accordingly, current OA management strategies aim to relieve symptoms, attenuate inflammation and promote cartilage regeneration. Pharmacological interventions, including systematic administration of anti-inflammatory corticosteroids or non-corticosteroids, can alleviate symptoms and improve quality of life. However, these approaches are noncurative, and their therapeutic efficacy is limited by the short half-life of the loaded cargos, which could be rapidly cleared by lymphatic or capillary drainage [6].

With the development of tissue engineering and nanotechnology, various kinds of microspheres were utilized in the fields of intra-articular injection and offered several advantages for OA treatment, including increased local drug concentration, enhanced bioavailability and reduced systemic side effects [7]. Microspheres can stabilize encapsulated agents and enable controlled release profiles, thereby minimizing toxicity and prolonging intra-articular retention of therapeutic cargos [8, 9]. In addition, microsphere-based delivery systems protect drugs from premature cellular uptake and enzymatic degradation while reducing drug from the articular space, collectively enhancing therapeutic efficacy and further limiting adverse effects of the loaded drugs [10].

Poly(d,l-lactide) (PDLLA) is a U.S. Food and Drug Administration–approved drug delivery carrier with excellent biocompatibility, and its degradation products are ultimately metabolized into water and carbon dioxide and excreted from the body [11]. Zatorska *et al*. [12] incorporated curcumin into PDLLA-based microspheres and proved that curcumin with a hydrophobic nature exhibits a strong affinity for the PDLLA matrix. In another study, a PDLLA-based composite hydrogel was developed for the controlled release of platelet lysate into the local microenvironment, showing prolonged intra-articular retention and favorable gelation properties, which underscored the delivery efficiency of platelet lysate–loaded PDLLA nanoparticles [13]. These results indicate that PDLLA-based microspheres are promising candidates for intra-articular injection in OA therapy [14]. In the present study, we intend to construct an injectable PDLLA-based microsphere with dual functionality for inflammation alleviation and cartilage regeneration.

To this end, kartogenin (KGN) and methylprednisolone hemisuccinate (MPHS) were incorporated into PDLLA/Polyvinyl Alcohol (PVA) microspheres. KGN is a small-molecule chondrogenic agent that has been shown to promote BMSCs chondrogenesis via activation of the PI3K-Akt signaling pathway and to exert protective effects on articular cartilage in animal models of OA [15–17]. However, the poor aqueous solubility of KGN limits its achievable concentration in solutions thereby reducing its therapeutic efficiency. As such, the development and optimization of KGN-loaded delivery systems are essential to enable effective intra-articular administration for OA treatment. Therefore, maintaining a sustained and near-linear release profile of KGN is critical to ensure continuous cartilage regeneration [18, 19], which is particularly suited for managing chronic diseases such as OA.

As a glucocorticoid, methylprednisolone suppresses inflammatory responses associated with severe allergic reactions, Coronavirus Disease 2019 (COVID-19), rheumatoid arthritis and OA [20, 21]. In particular, intra-articular injection of MPHS has been shown to effectively relieve pain, a primary clinical symptom of OA [22]. However, frequent administration of glucocorticoids can lead to serious adverse effects, including hormone dependence, gastrointestinal hemorrhage, bone loss and respiratory complications [21, 23]. Encapsulating MPHS within a carrier system and enabling its controlled release into the local microenvironment over a relatively short duration may mitigate OA-associated inflammation while minimizing systemic toxicity. Therefore, an initial burst release of MPHS followed by rapid attenuation of drug release may provide effective inflammation relief while reducing the risk of glucocorticoid-related side effects.

In this study, a novel dual-releasing system was constructed to deliver KGN and MPHS in a coordinated and controlled manner. As the microspheres degraded from the top down, MPHS encapsulated in the outer shell of the microspheres eluted faster than core-loaded KGN, thereby enabling sequential drug delivery.

## Materials and methods

### Fabrication of the KGN/MPHS-loaded microspheres

**Figure 1:**
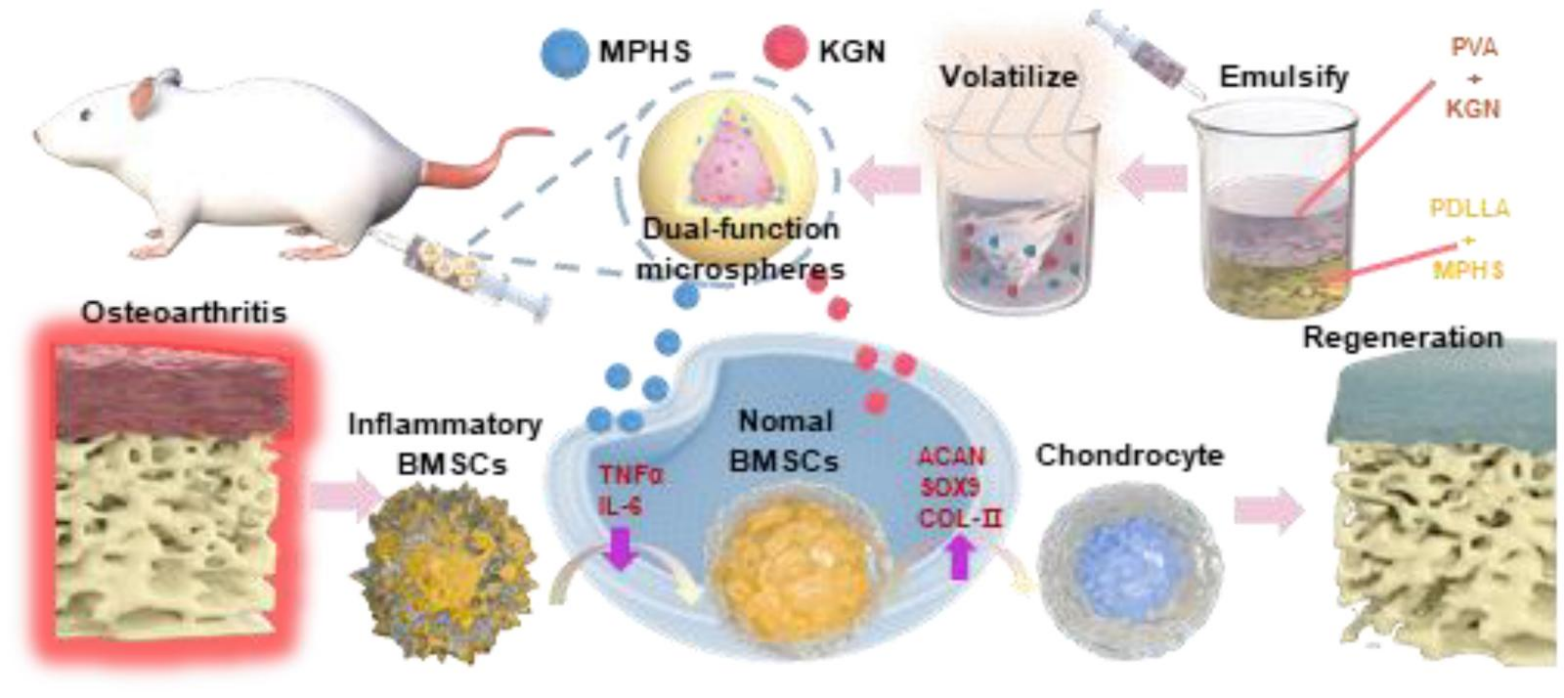
Schematic illustration of the fabrication process of the MS_KM_ microspheres.

The KGN-loaded (MS_K_), MPHS-loaded (MS_M_), KGN/MPHS-loaded (MS_KM_) and blank (MS_B_) microspheres were synthesized using the emulsification-solvent evaporation technology (Figure 1). Briefly, 600 mg PDLLA and 20 mg MPHS were dissolved in 16 mL dichloromethane and stirred for 30 min. A total of 5 mg KGN and 3 g PVA were dissolved in 150 mL ddH_2_O and stirred for 10 min. The MPHS/PDLLA solution was added dropwise to the KGN/PVA aqueous solution to form an oil-water mixture and emulsified for 5 min using a sonicator (SCIENTZ Biotech, Ningbo, China), and then gently stirred for 12 h to evaporate dichloromethane in the emulsion. After dialysis with deionized water 3 times, the microspheres were lyophilized for further use (Figure 1).

### Characterization of the microspheres

The Fourier transform infrared spectroscopy (FTIR) spectra of the synthesized MS_K_, MS_M_, MS_KM_ and MS_B_ were obtained using a Nicolet 170-SX spectrophotometer (Thermo Nicolet Ltd., USA) with a frequency range of 4000–400 cm^−1^. X-ray photoelectron spectroscopy (XPS) was performed to determine the surface chemical compositions of the MS_B_, MS_M_ and MS_KM_ by an Axis Ultra DLD apparatus (Kratos, UK). Crystalline features corresponding to PDLLA and PVA were analyzed by X-ray diffraction (XRD, X′ Pert Pro, The Netherlands). The morphology of dried MS_KM_ was examined by a field-emission scanning electron microscopy (FE-SEM; Zeiss Sigma, Germany) after gold sputter coating. A total of 10 μL of MS_KM_ suspension was deposited onto a copper grid, air-dried and imaged using a JEM-2110 microscope (JEOL, Tokyo, Japan). The particle size distribution of MS_KM_ was measured using a laser particle size analyzer (Mastersizer 2000, Malvern Instruments Ltd, UK). The core-shell structure of MS_KM_ was further visualized by fluorescence microscope (Nikon Eclipse 80i, Japan) after loading rhodamine B-labeled MPHS and calcein-labeled KGN.

### Biocompatibility of the microspheres

The protocol of the *in vivo* experiment was approved by the Wuhan University’s Animal Care and Use Committee (No. S07920120C). Rat bone marrow stromal cells (MSCs) were isolated from femurs of SD rats and cultured in DMEM supplemented with 10% fetal bovine serum (FBS), 100 U/mL streptomycin and 100 U/mL penicillin as previously described [24]. Briefly, bone marrow was flushed out from both sides of the rat femur and repeatedly pipetted by a sterile syringe. Then, the marrow solution was centrifuged, resuspended in fresh DMEM supplemented with 10% FBS, 100 U/mL streptomycin and 100 U/mL penicillin and cultured at 37°C, 5% CO_2_. After 24 h of incubation, non-adherent cells were removed by the first change of culture medium.

After reaching 80% confluence, the isolated MSCs were re-expanded and co-cultured with the MS_K_, MS_M_, MS_KM_ and MS_B_, and their viability was evaluated by cell counting kit-8 (CCK-8) assay at 1, 3, 5 and 7 days of incubation. Briefly, a certain amount of microspheres (5 mg/mL) and MSCs were co-cultured with 90 μL culture medium (DMEM supplemented with 10% FBS, 100 U/mL streptomycin and 100 U/mL penicillin) and 10 μL CCK-8 reagent in a 96-well culture plate. After 1 h of incubation at 37°C, 5% CO_2_, the absorbance was measured by a microplate reader at 450 nm.

Live/Dead staining was also performed to evaluate the effects of MS_K_, MS_M_, MS_KM_ and MS_B_ on cell proliferation. According to the instructions provided by the manufacturer of the LIVE/DEAD kit (Invitrogen, Carlsbad, CA, USA) [25], a mixture of 4 mM calcein AM and 2 mM ethidium homodimer was incubated with microspheres and BMSCs for 15 min at 37°C, 5% CO_2_. Inoculation of BMSCs onto a 6-well culture plate was treated as a control. After rinsing with phosphate buffer saline (PBS) 3 times, cells in all groups were observed by fluorescence microscopy (Nikon, Japan). The live cells exhibit a green color, whereas the red color indicates the dead cells without esterase activity.

### Release profiles of KGN and MPHS from MS_KM_

The release profiles of KGN and MPHS from MS_KM_ were tested in PBS. Firstly, 20 mg of lyophilized MS_KM_ was uniformly dispersed in 30 mL of PBS (pH = 7.4) and incubated at 37°C under horizontal shaking for 1, 3, 6, 10, 15, 25 and 40 days. At each time point, the suspension was centrifuged at 8000 rpm for 10 min, and the concentrations of KGN and MPHS in 3 mL of the collected supernatant were quantified. An equal volume of fresh PBS was then added to redisperse the microspheres for continued incubation. Cumulative release of KGN and MPHS was then calculated by Ultraviolet-visible spectroscopy at the given times. The cumulative release percentage was calculated by summing the amounts of KGN and MPHS released at each interval and dividing by the total amount of encapsulated drug. The morphology of MS_KM_ after drug release was also observed using SEM.

### Safranin O staining

To investigate chondrogenic differentiation of BMSCs in a 2D culture mode, Safranin O staining was then performed to detect the accumulation of proteoglycan during the chondrogenic differentiation of BMSCs in a 2D culture mode. Briefly, 2.5 × 10^5^ cells were inoculated onto the 6-well culture plate and then cultured with the chondrogenic induction medium, which consisted of DMEM/F-12 supplemented with bovine serum albumin (BSA) (7.5% w/v), insulin-transferrin-selenium (1% v/v), dexamethasone (10^−7^ M), ascorbate-2-phosphate (50 µg/mL), L-proline (50 µg/mL) and sodium pyruvate (1 mM). Interleukin-1β (IL-1β) (10 ng/mL) was then added into the culture medium to induce the inflammatory process of BMSCs for 24 h after 7 days of chondrogenic induction. After 2 and 4 weeks of incubation, Alcian blue staining was finally utilized to detect the effects of the MS_K_, MS_M_, MS_KM_ and MS_B_ on the proteoglycan expression.

### Alcian blue staining

Alcian blue staining was performed to detect GAGs expression associated with chondrogenic differentiation of BMSCs pellets in a 3D culture mode. Briefly, 2.5 × 10^5^ cells were centrifuged for 10 min at 1780 rpm to transform into the pellets and cultured in chondrogenic induction medium consisting of DMEM/F12 (Gibco, USA), 1% penicillin G-streptomycin (Sigma, USA), 1% ITS Media Supplement (Beyotime, China), 1.25 mg/mL BSA (Solarbio, China), 1 mM sodium pyruvate (Solarbio, China), 10^−7^ M dexamethasone (Sigma, USA), 50 µM ascorbate 2-phosphate (Sigma, USA), 50 µM L-proline (Solarbio, China), and 10 ng/mL TGF-β1 (Solarbio, China). After 21 days of chondrogenic differentiation, IL-1β (10 ng/mL) was utilized and added into the culture medium as a stimulation factor to induce the *in vitro* aseptic inflammation of BMSCs pellets for 24 h [26]. After IL-1β treatment, BMSCs pellets were co-cultured with the MS_K_, MS_M_, MS_KM_ and MS_B_ and then subjected to the subsequent experiments. The normal culture media with no microspheres in the control were utilized to culture IL-1β-induced BMSCs pellets. An equal amount of BMSCs pellets cultured with normal culture media without the addition of IL-1β was treated as a sham group. After 2 and 4 weeks of incubation, BMSCs pellets were fixed in 4% PFA for 2 h, immersed in 20% sucrose for 2 h, embedded in Tissue-Tek O.C.T. Compound (Sakura Finetek, Japan) at −80°C for 10 min, and sectioned on a cryotome (Leica, Germany) into sections of 5 µm. Alcian blue staining was then utilized to detect the effects of various kinds of microspheres on the proteoglycan expression using an Alcian Blue Stain Kit (Solarbio, China) according to the previous protocol [27].

### Transwell assay

The chemotactic effects of MS_K_, MS_M_, MS_KM_ and MS_B_ on BMSCs were evaluated by a Transwell assay using a Transwell chamber with 8 µm pores (Corning Costar, Cambridge, MA, USA). Briefly, the upper chamber was seeded with BMSCs (3 × 10^5^ cells), which were cultured with the serum-free culture media. A total of 10 mg of MS_K_, MS_M_, MS_KM_ and MS_B_ were added into the lower chamber of each well. Also, the serum-free media with no microspheres was used as a control. After 12 h of incubation at 37°C, 5% CO_2_, the migrated cells were fixed by 4% paraformaldehyde for 15 min and stained with crystal violet solution (0.5% w/v, Macklin, China). After gently removing non-migrated cells by a cotton swab, the migrated BMSCs in five random fields were imaged, counted, and averaged through a bright-field microscope.

### RT-PCR

Real-time PCR was used to detect the expression of several chondrogenic differentiation-related marker genes (Sox 9, Col-II, ACAN) at 7 and 21 days, respectively. For chondrogenic differentiation, BMSCs were centrifuged into pellets (3 × 10^5^ cells/pellet) in 15 mL centrifuge tubes and cultured in chondrogenic medium with each type of microsphere or without microspheres. After 7 and 21 days, the pellets were mechanically crushed using a high-throughput tissue grinder (Scient Z, China), and total RNA was extracted using RNAiso Plus (Takara, Japan).

To evaluate the anti-inflammatory potential of the microspheres, an *in vitro* model was used consisting of BMSCs stimulated with IL-1β [28]. BMSCs were seeded at a density of 1 × 10^6^ cells/mL in 6-well plates and incubated for 24 h. Then, the medium was changed with each type of microspheres or without microspheres. After 1 and 3 days, the cells were collected to evaluate the expression of inflammatory genes (TNF-α and IL-6).

Total RNA was extracted using RNAiso Plus according to the manufacturer’s instructions, and its concentration was determined using an ultramicro-spectrophotometer (Denovix, USA). Then, reverse transcription was performed according to the manufacturer’s protocol using the PrimeScript RT reagent Kit (Takara, Japan). Amplification and detection were performed using the TB Green^®^ Premix Ex Taq™ II (Tli RNaseH Plus) (Takara, Japan) and the StepOnePlus real-time PCR system (Applied Biosystems, USA) with the following profile: 1 cycle at 95°C for 30 s and 40 cycles each at 95°C for 5 s and 60°C for 30 s. GAPDH was used as a housekeeping gene, and the relative expression was determined by the 2^−ΔΔCT^ method. For each gene, triplicates of three different experiments were carried out. Primers (Sangon Biotech, China) used are listed in Table S1.

### Elisa

BMSCs were seeded at a density of 1 × 10^6^ cells/mL in 6-well plates and incubated for 24 h. Then, the medium was changed with each type of microsphere or without microspheres. After 1 and 3 days, the concentration of the cytokines TNF-α and IL-6 in the medium after being in contact with the microspheres was evaluated. For that, the Rat TNF-alpha ELISA kit (Beyotime, China) and Rat IL-6 ELISA Kits (Beyotime, China) were used according to the manufacturers’ instructions. The absorbance value was read at 450 nm in a microplate reader (Molecular Devices, USA). The corresponding concentration of the sample was calculated by the absorbance values and the standard curve.

### Immunofluorescence staining for Col-II

The collagen type-II (Col-II) expression level of BMSCs pellets in MS_K_, MS_M_, MS_KM_ and MS_B_ groups was analyzed by immunofluorescence staining [29]. Also, BMSCs pellets cultured with normal culture media with and without the addition of IL-1β were treated as control and sham groups, respectively. After 2 and 4 weeks of culture, the BMSCs pellets were embedded in frozen section media and sectioned into slices through a frozen slicer (Leica CM 1950). After being immobilized with 4% paraformaldehyde, the slides with a thickness of 6 μm were rinsed with PBS 3 times, permeabilized with 1% Triton X-100, and then treated with 2% BSA for 1 h to block the nonreaction sites. The slides were incubated with the primary antibody against Col-II (1:200, Rb anti-Ms, Affinity Biosciences, China) overnight at 4°C and then incubated with CoraLite^®^488-labeled secondary antibodies (1:100, Gt anti-Rb, Proteintech, USA) for 2 h at room temperature. After being counterstained with 4′,6′-diamidino-2-phenylindole (DAPI) for 15 min, the samples were observed and photographed through a fluorescence microscope (ZEISS, Germany).

### Animal experiment

A total of 40 SD male rats were divided into five groups randomly: sham, OA and MS_K_, MS_M_, MS_KM_ (*n* = 8). The rats in the MS_K_, MS_M_, MS_KM_ and OA groups were injected with 100 μL monosodium iodoacetate (MIA) solution (30 mg/mL) into the right side of the knee joint to establish the OA model [30]. Intraarticular injection of 100 μL of 0.9% saline solution was used as a sham group. After 7 days of MIA treatment, all the rats were treated with MS_K_, MS_M_, or MS_KM_ by intraarticular injection (9.45 mg/kg) [31, 32]. After 4 and 8 weeks of IA injection, rats were euthanized by inhalation of carbon dioxide. The distal femora in each group were fixed in 4% paraformaldehyde for 48 h, soaked in 10% ethylenediaminetetraacetic acid (EDTA) for 8 weeks to complete decalcification, dehydrated with a series of graded alcohol concentrations, and embedded into paraffin wax. Sagittal sections of the femoral bones with the thickness of 4 μm were performed with Safranin O/Fast green staining to analyze the expression of glycosaminoglycans (GAGs) [33]. After that, immunohistochemistry of Col-II, aggrecan (Agg), and interleukin-1 (IL-1) was performed by blocking the nonspecific sites using 3% BSA and staining paraffin sections with the primary rabbit anti-Col-II (1/100, Abcam, Cambridge, UK), rabbit anti-aggrecan (1/100, Abcam, Cambridge, UK), and anti-IL-1 antibodies (1:400; Cell Signaling Technology, Beverly, MA) at 4°C overnight. Then, the sections were incubated with the secondary goat anti-rabbit antibodies (Servicebio, Wuhan, China). Finally, color was developed using 3, 3-diaminobenzidine tetrahydrochloride (DAB, Servicebio, Wuhan, China) system and counterstained with hematoxylin. The internal organ toxicity (heart, liver, spleen, lung and kidney) was also analyzed by HE staining.

Moreover, the retention profile of MS_KM_ was analyzed in this study. Briefly, a total of 20 C57BL/6 male mice at the age of 4 weeks were randomly divided into 2 groups: Rhodamine B-labeled MS_KM_ and free injection groups (*n* = 5). According to the above-mentioned protocol, 1 mg of Rhodamine B was added into KGN/PVA aqueous solution and stirred for 10 min. The MPHS/PDLLA solution was added dropwise to the Rhodamine B/KGN/PVA solution, emulsified for 5 min, and gently stirred for 12 h to form the Rhodamine B-labeled MS_KM_. A total of 50 μL of Rhodamine B-labeled MS_KM_ solution (9.45 mg/kg) was then administered into the knee joints of each animal. Intra-articular injection of Rhodamine B/KGN/PVA blend solution was used as a free injection group. Briefly, 1 mg of Rhodamine B, 20 mg of MPHS, and 5 mg of KGN were dissolved in 150 mL ddH_2_O and stirred for 10 min. Then, 50 μL of blend solution was injected into the right knee joint of each animal. After 1 h, 14 days and 28 days of administration, the mice were anesthetized by isoflurane inhalation and subjected to IVIS.

## Results and discussion

### Fabrication and characterization of MS_KM_ microspheres

**Figure 2:**
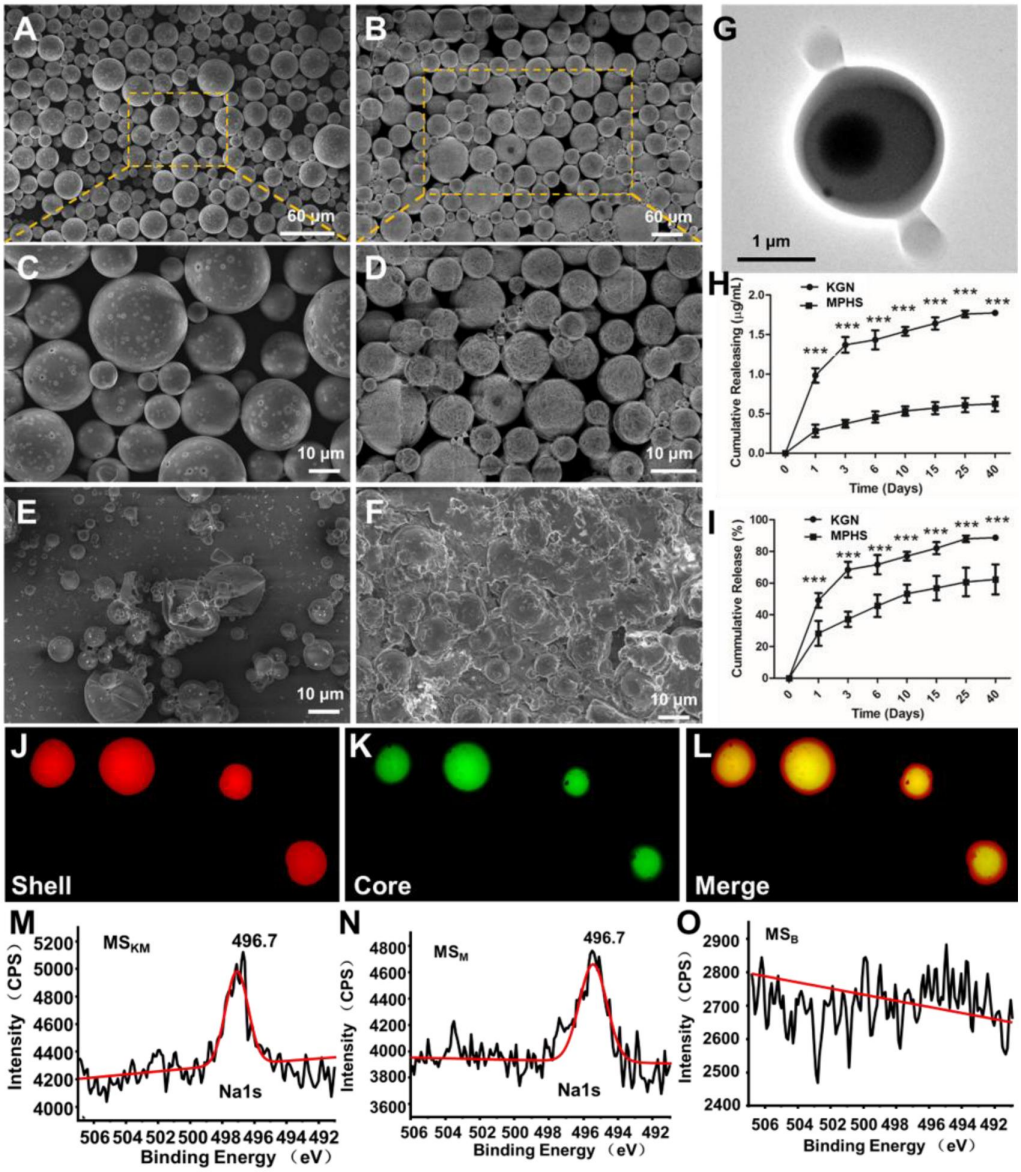
Preparation and characterization of microspheres. SEM images of the MS_KM_ after (**A**, **C**) 0, (**B**, **D**) 3, (E) 10 and (F) 25 days of incubation in PBS. Scale bars represent 60 and 10 μm. The insert images represent the respective high-magnification images. (**G**) TEM image of the MS_KM_. (**H** and **I**) *In vitro* releasing profiles of KGN and MPHS from the MS_KM_. Data are presented as mean ± SD. *P < 0.05, **P < 0.01, ***P < 0.001. Scale bars represent 1 μm. (**J**–**L**) Core-shell structure of the MS_KM_. XPS spectra of the (**M**) MS_KM_, (**N**) MS_M_ and (**O**) MS_B_.

The morphology of MS_KM_ was examined by SEM, and the results were shown in Figure 2. MS_KM_ exhibited a spherical morphology with a porous surface (Figure 2A and C). Furthermore, MS_KM_ exhibits a typical core-shell structure (Figure 2L), in which KGN (green fluorescence, Figure 2K) is encapsulated in the core, while MPHS (red fluorescence, Figure 2J) is distributed in the shell. To further verify the composition of MS_K_, MS_M_, MS_KM_ and MS_B_, XPS analysis was used to detect peaks from Na, a characteristic element of MPHS. As shown in Figure 2, the typical peak 496.7 eV from sodium (Na1s) could be detected in both MS_KM_ and MS_M_, confirming the successful incorporation of MPHS into MS_KM_ and MS_M_ (Figure 2J–L).

**Figure 3:**
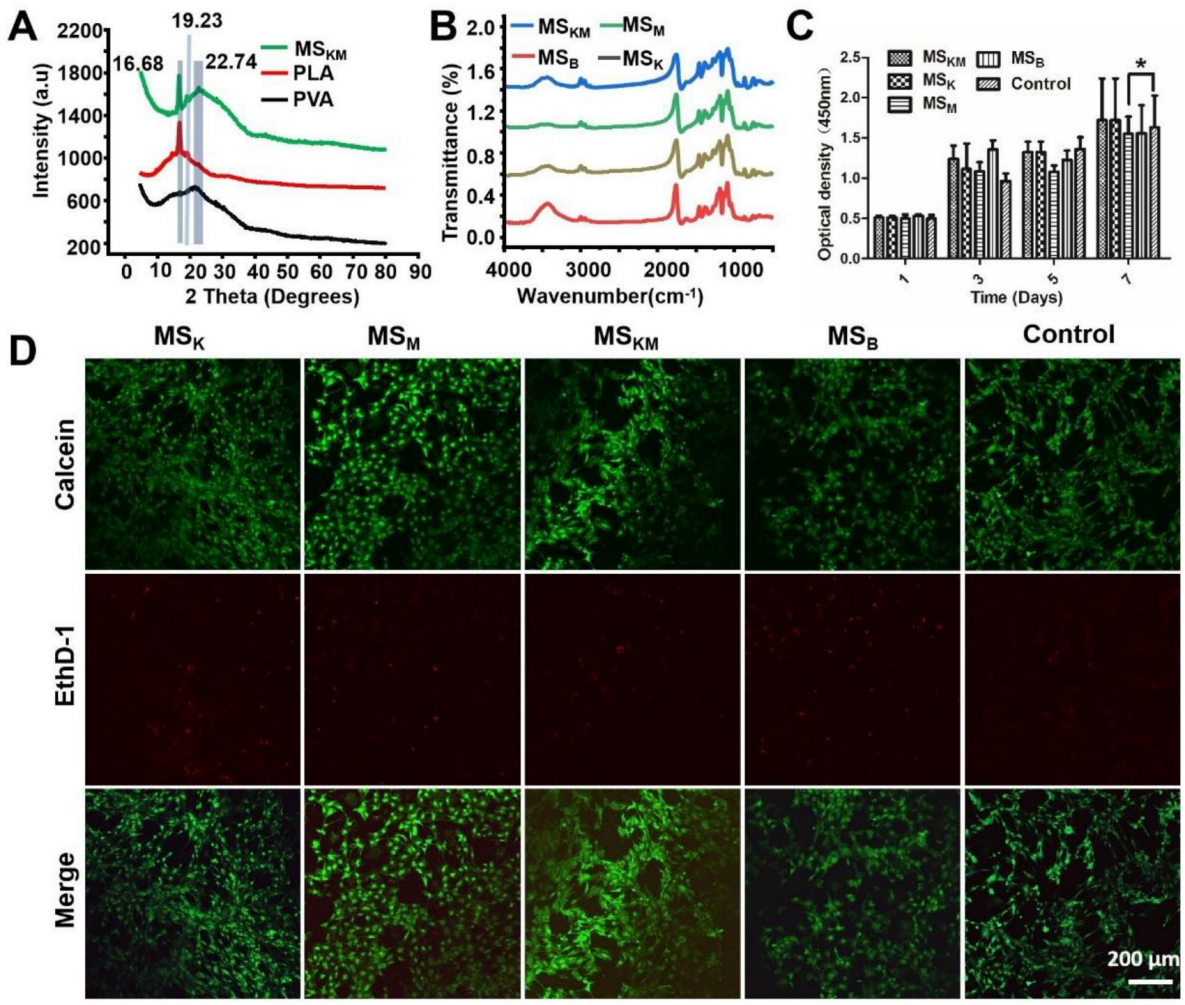
Characterization and biocompatibility of microspheres. (**A**) XRD analysis of the MS_KM_, PLA and PVA. (**B**) FTIR analysis of the MS_KM_, MS_M_, MS_K_ and MS_B_. (**C**) Viability of BMSCs was analyzed through CCK-8 test. Data are presented as mean ± SD. *P < 0.05, **P < 0.01, ***P < 0.001. (D) LIVE-DEAD staining of BMSCs cocultured with the MS_KM_, MS_M_, MS_K_ and MS_B_. Scale bars represent 200 μm.

The crystalline structures of the MS_KM_, PDLLA and PVA were analyzed by XRD, and the corresponding results are presented in Figure 3. The diffraction peak at 22.74° corresponds to the characteristic peak of PVA [34] and was observed in both the commercial PVA and MS_KM_ samples. Additionally, the PDLLA and MS_KM_ samples exhibited a diffraction peak at 16.8° (Figure 3A), corresponding to the (110/200) planes of PDLLA [35]. A sharp peak at 2θ = 19.23°, assigned to the (010) plane of PDLLA, was also present in both PDLLA and MS_KM_, further demonstrating the successful incorporation of PDLLA into MS_KM_. These XRD results indicate that PDLLA and PVA are immiscible and that both components were successfully encapsulated within MS_KM_.

FTIR spectroscopy was subsequently employed to analyze the functional groups of the samples. The characteristic absorption peaks of MS_K_, MS_M_, MS_KM_ and MS_B_ were similar to each other (Figure 3B), indicating that no new functional groups were generated during microsphere fabrication and after the incorporation of KGN and MPHS.

### Releasing profiles of KGN and MPHS from MS_KM_ microspheres *in vitro*

The accumulative release of KGN and MPHS from MS_KM_ in PBS was investigated to assess the dual-functional performance of microspheres. Both KGN and MPHS from MS_KM_ displayed a typical biphasic release profile, characterized by an initial burst release stage within the first 1–3 days, followed by a constant release phase thereafter (Figure 2H). Among them, KGN showed a longer burst-release period of ∼3 days, compared with the more rapid burst release of MPHS within 1 day (Figure 2H). This difference can be attributed to the spatial distribution of the two drugs within MS_KM_: KGN was encapsulated in the core and was released in a slower and more sustained manner due to the combined diffusion barriers imposed by the PDLLA matrix and the outer shell layer. The transition from the burst-release phase to the sustained-release phase was clearly observed for both KGN and MPHS, indicating a well-regulated release behavior (Figure 2H). Meanwhile, the cumulative release of KGN from MS_KM_ reached ∼88.69 ± 1.70%, which was significantly larger than the cumulative release percentage of MPHS (62.34 ± 9.45%) during 40 days of incubation (*P* < 0.001, Figure 2I). The lower cumulative release of MPHS may be attributed to partial drug loss during microsphere fabrication, as MPHS was primarily distributed within the shell layer of MS_KM_.

The degradation behavior of MS_KM_ was further examined by SEM. As shown in Figure 2, the overall morphology of MS_KM_ remained relatively intact during the initial 3 days of incubation in PBS (Figure 2B). Small surface pores were observed, resulting from gradual PDLLA hydrolysis into oligomers, as illustrated in the enlarged image (Enlarged picture, Figure 2D). During this period, MS_KM_ maintained a relatively uniform particle size and morphology (Figure 2B). In contrast, after 10 days of incubation, the structural integrity of MS_KM_ began to deteriorate, as evidenced by a roughened, cracked surface and a noticeable reduction in both particle size and number (Figure 2E). With continued PDLLA degradation and drug release, MS_KM_ developed an increasing number of surface pores and progressively dissolved, ultimately leading to structural collapse after 25 days of incubation (Figure 2F). Consistent with these morphological changes, the plateau observed in the release curves of KGN and MPHS indicates that drug release was largely completed by this time point, corresponding to the collapse of the MS_KM_ structure.

### Biocompatibility of the MS_KM_ microspheres

The cytotoxicity of the MS_K_, MS_M_, MS_KM_ and MS_B_ on BMSCs was assessed through a CCK-8 assay and Live/Dead staining. The CCK-8 assay results indicated a steady increase in the number of BMSCs over time, with no significant difference observed among the MS_K_, MS_M_, MS_KM_ and MS_B_ groups at 1, 3, 5 and 7 days of co-culture (*P* > 0.05, Figure 3C). This suggests that MS_K_, MS_M_, MS_KM_ and MS_B_ have no positive or negative effect on cell proliferation.

The results of Live/Dead staining were basically consistent with the CCK-8 assay. As shown in Figure 3D, the majority of BMSCs kept alive in all groups, and almost no dead cells was observed after 48 h of incubation (Figure 3D). Collectively, it is safely concluded from the results of the CCK-8 assay and Live/Dead staining that the biocompatibility of the MS_K_, MS_M_, MS_KM_ and MS_B_ was excellent, which is favorable for the *in vitro* culture of cells and a precondition for the *in vivo* experiments.

### The expression of GAGs analyzed by safranin O and Alcian blue staining

**Figure 4:**
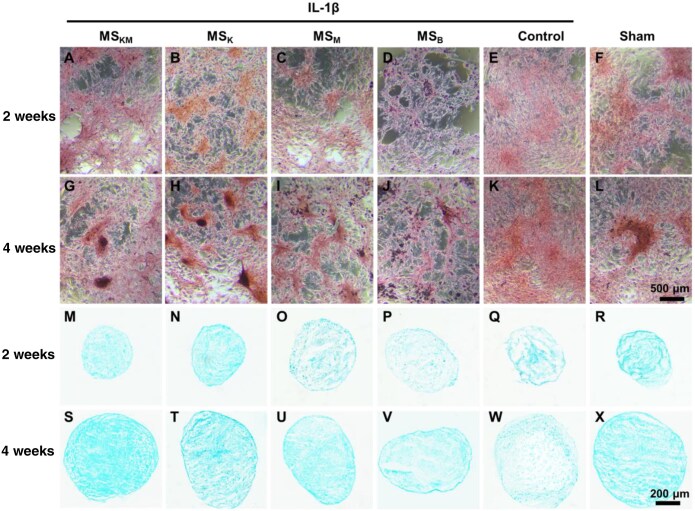
The chondrogenic differentiation of BMSCs pellets cocultured with the MS_KM_, MS_K_, MS_M_ and MS_B_ in a 3D culture mode. (**A**–**L**) Safranin O staining of BMSCs in a 2D culture mode after 2 and 4 weeks of *in vitro* culture. Scale bars represent 500 μm. (**M**–**X**) Alcian blue staining of BMSCs pellets in a 3D culture mode after 2 and 4 weeks of *in vitro* culture. Scale bars represent 200 μm.

Safranin O staining and Alcian blue staining were conducted to analyze the chondrogenic effects of MS_KM_, MS_K_, MS_M_ and MS_B_ on IL-1β-induced BMSCs in 2D and 3D culture, respectively. It could be observed that the intensity of Safranin O staining was strongest in the MS_K_ group, followed by the MS_KM_ and Blank groups. These results of Safranin O staining reflect the proteoglycan accumulation in BMSCs, in the order of MS_K_>MS_KM_>MS_B_, after 2 and 4 weeks of culture (Figure 4A–L). Compared with the Sham group, chondrogenesis of BMSCs was significantly enhanced in both the MS_KM_ and MS_K_ groups, which is a favorable improvement due to the sustained release of KGN from MS_KM_ and MS_K_ (Figure 4A–L).

The above-mentioned results were basically confirmed with that of Alcian blue staining. The chondrocyte pellets in both the MS_KM_ and MS_K_ groups displayed the strongest staining intensity after 2 weeks of culture, which is comparable with that of the sham group (Figure 4M, N, R). At 4 weeks of culture, the staining intensity of the MS_KM_ group still accounted for the highest among all the groups (Figure 4S–X). However, the staining intensity of the blue color in the MS_M_ group was increased and attained a similar level with that of the MS_K_ group with increasing induction time (Figure 4T and U). Compared with the sham group, the expression of GAGs in the MS_B_ and control groups was significantly lower due to the negative influence of IL-1β on the chondrogenic differentiation of BMSCs (Figure 4V–X).

### The influence of MS_KM_ on cells migration and chondrogenic differentiation abilities

**Figure 5:**
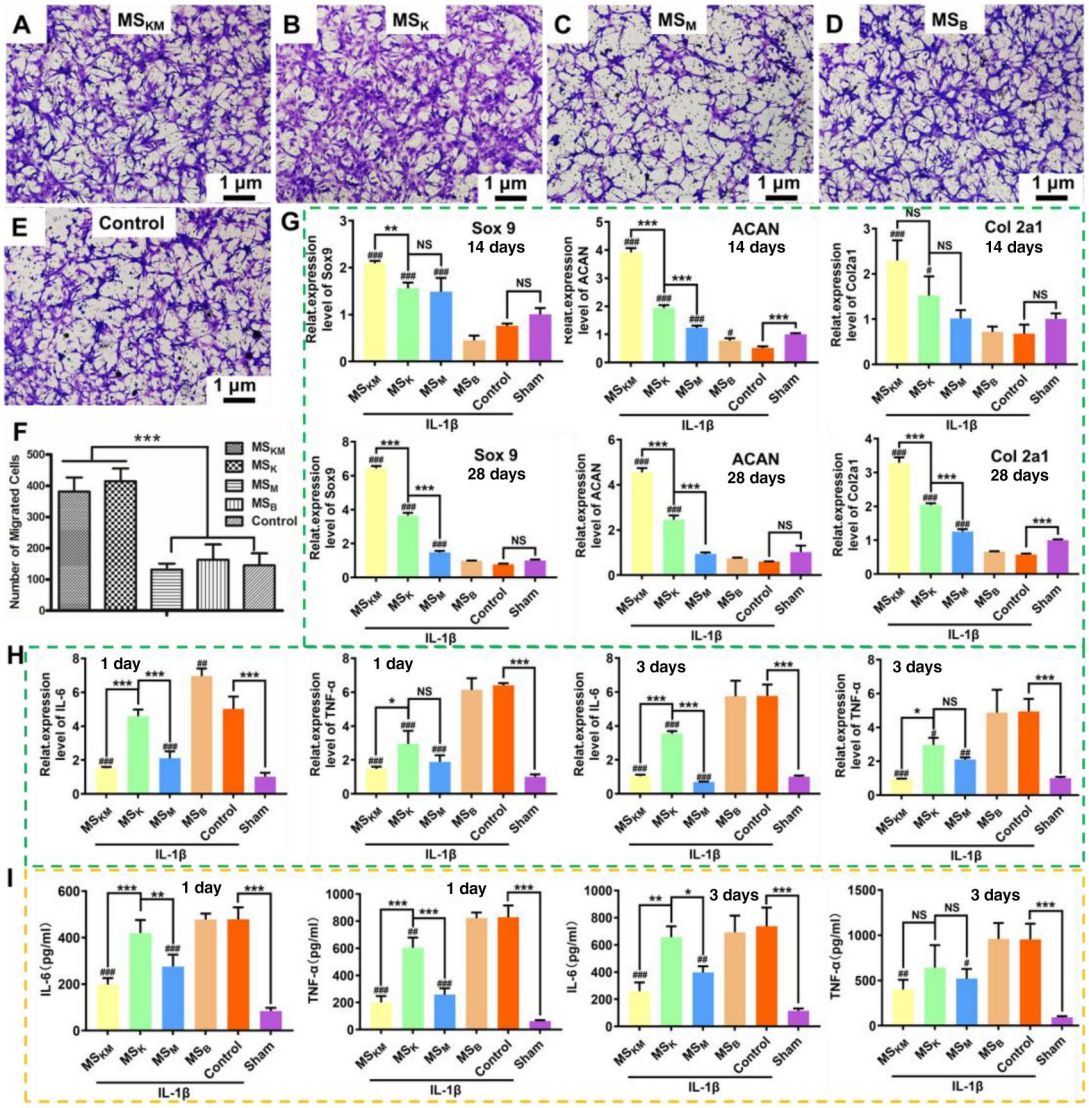
(**A**–**F**) Migration of BMSCs treatment with MS_KM_, MS_M_, MS_K_ and MS_B_ was analyzed by Transwell assay. Scale bars represent 100 μm. (**G**) The gene expression of chondrogenic factors were evaluated using RT-PCR. (**H**) The gene expression of inflammation factors was evaluated using RT-PCR. (**I**) The protein expression of inflammation factors was evaluated using ELISA. Data are presented as mean ± SD. *P < 0.05, **P < 0.01, ***P < 0.001.

The migration ability of BMSCs influenced by microspheres was evaluated by Transwell assay. The number of the migrated cells in the MS_KM_ and MS_K_ groups was significantly larger than those of the MS_M_, MS_B_ and control groups (Figure 5A–F, *P* < 0.001), indicating that KGN has a chemotactic effect on the migration of BMSCs.

The gene expression of chondrogenic factors, including Sox 9, Aggrecan (ACAN), and Col2a1 were evaluated using RT-PCR. Our aim was to assess the effects of MS_KM_, MS_K_, MS_M_ and MS_B_ microspheres on chondrogenic differentiation and inflammation relief. Notably, ACAN and Col2a1, key components of the cartilage matrix [36], showed significant changes following treatment. The expression of Sox 9 is notably high in differentiating chondrocytes and plays a pivotal role in chondrogenesis [37]. Specifically, IL-1β treatment resulted in a notable suppression of ACAN expression in the MSB group as compared to the Sham group after 14 days of *in vitro* culture (Figure 5G, *P* < 0.05), demonstrating the negative influence of inflammation on BMSC chondrogenesis. As expected, the addition of MS_B_ had limited efficacy in increasing mRNA levels of Sox 9, Col2a1 and ACAN, which were similar to the results of the control group after both 14 and 28 days of culture (Figure 5G). In contrast, the MS_K_ group displayed a significant upregulation in ACAN and Col2a1 expression (*P* < 0.05), underscoring its positive role in cell chondrogenesis. By contrast, the gene expression levels of Sox 9, ACAN and Col2a1 in the MS_KM_ and MS_K_ groups were markedly higher than in the MS_B_ and sham groups at all time points (Figure 5G, *P* < 0.05), highlighting the superior efficacy of MS_KM_ in enhancing chondrogenic differentiation of BMSCs. Notably, the gene expression of Sox 9, ACAN and Col2a1 in BMSCs treated with MS_KM_ microspheres were higher than those in the MS_K_ group at all time points (*P* < 0.05). Collectively, these results revealed that MS_KM_ and MS_K_ are effective in enhancing chondrogenic differentiation of BMSCs, and a synthetic release of KGN and MPHS effectively promotes the chondrogenic differentiation of BMSCs.

**Figure 6:**
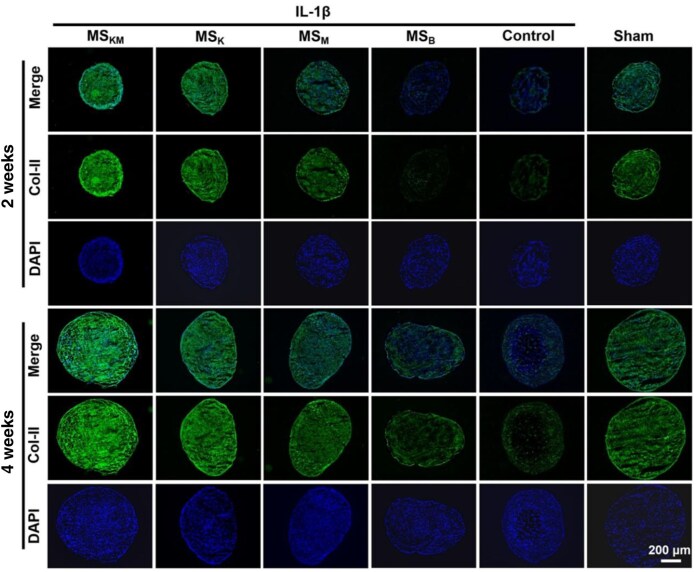
Immunofluorescence staining of Col-II in BMSCs pellets after 2 and 4 weeks of *in vitro* coculture with IL-1β. Scale bars represent 200 μm.

Furthermore, an immunochemical assay was performed to analyze the protein expression of Col-II, a typical biomarker of cartilage matrix [36]. As illustrated in Figure 6, a notable decrease in the protein expression level of Col-II was observed following IL-1β treatment. This was evidenced by reduced green fluorescence intensity in the control group, as opposed to the sham group, after 2 weeks of co-culture (Figure 6). MS_B_-treated BMSCs showed minimal Col-II expression (Figure 6), whereas both MS_KM_ and MS_K_ treatments led to a significant increase, corroborating the results of Alcian blue (Figure 4). Additionally, in the MS_M_ group, Col-II expression in BMSC pellets treated with IL-1β and MS_M_ was superior to the MS_B_ group (Figure 6), suggesting MPHS’s positive role in promoting cartilage regeneration.

After an additional 2 weeks of co-culture, fluorescence intensity and size of BMSCs pellets in the MS_KM_ group remained the largest among all groups at 4 weeks (Figure 6). A noticeable increase in Col-II expression was observed in the MS_K_ group compared to MS_M_ and MS_B_ (Figure 6), highlighting KGN’s role in chondrogenesis. Notably, Col-II expression in the MS_KM_ group was the highest at 4 weeks of co-culture (Figure 6), reinforcing the synergistic effect of KGN and MPHS in chondrogenesis.

### The expression of inflammation markers

The expression of inflammatory proteins, including IL-6 and TNF-α, in BMSCs treated with IL-1β followed by co-incubation with MS_KM_, MS_M_, MS_K_ and MS_B_ was assessed using RT-PCR and ELISA. IL-1β treatment for 1 day led to an elevated mRNA expression of inflammatory markers IL-6 and TNF-α (the control versus the Sham group) (Figure 5H, *P* < 0.05). Conversely, MS_KM_ and MS_M_ treatments resulted in a notable decrease in these inflammatory markers, indicating effective inflammation mitigation (Figure 5H, *P* < 0.05). Noteworthy, MS_K_-treated BMSCs showed intermediate levels of IL-6 and TNF-α expression (Figure 5H, *P* < 0.05), suggesting a partial suppression of inflammation in MS_K_-treated BMSCs. This suggests that the sustained release of KGN not only promotes cartilage formation but also partially suppresses the gene expression of inflammatory factors.

These results were largely consistent with ELISA findings, further validating the effectiveness of MS_KM_ and MS_M_ in reducing inflammation in IL-1β-induced BMSCs. Both the MS_B_ and control groups displayed higher IL-6 and TNF-α protein expression than the sham group (Figure 5I, *P* < 0.05), confirming the validity of the IL-1β-induced BMSC-based *in vitro* inflammation model. The inflammation in BMSCs treated with MS_KM_ and MS_M_ microspheres was significantly reduced (Figure 5I), demonstrating that sustained release of MPHS mitigates the inflammation in IL-1β-induced BMSCs. However, the MS_K_ group still showed higher IL-6 and TNF-α protein expression compared to the MS_KM_ and MS_M_ groups (Figure 5I, *P* < 0.05). Furthermore, the protein expression levels of IL-6 and TNF-α in BMSCs were nearly identical to those in the control and MS_B_ groups (Figure 5I, *P* > 0.05). This indicates that KGN’s sustained release alone is insufficient for reducing inflammation, highlighting the need for a synergistic release of KGN and MPHS. After three days of IL-1β treatment, the protein expression of IL-6 and TNF-α in the MS_B_ and control groups remained higher than in the sham group (*P* < 0.05). The IL-6 protein expression in the MS_KM_ group was significantly lower than that in the MS_K_ group (Figure 5I, *P* < 0.05) and almost identical to that in the MS_M_ group (Figure 5I, *P* < 0.05), affirming that the combined sustained release of KGN and MPHS is an effective strategy for mitigating inflammation in BMSCs treated with IL-1β.

### *In vivo* retention of MS_KM_

**Figure 7:**
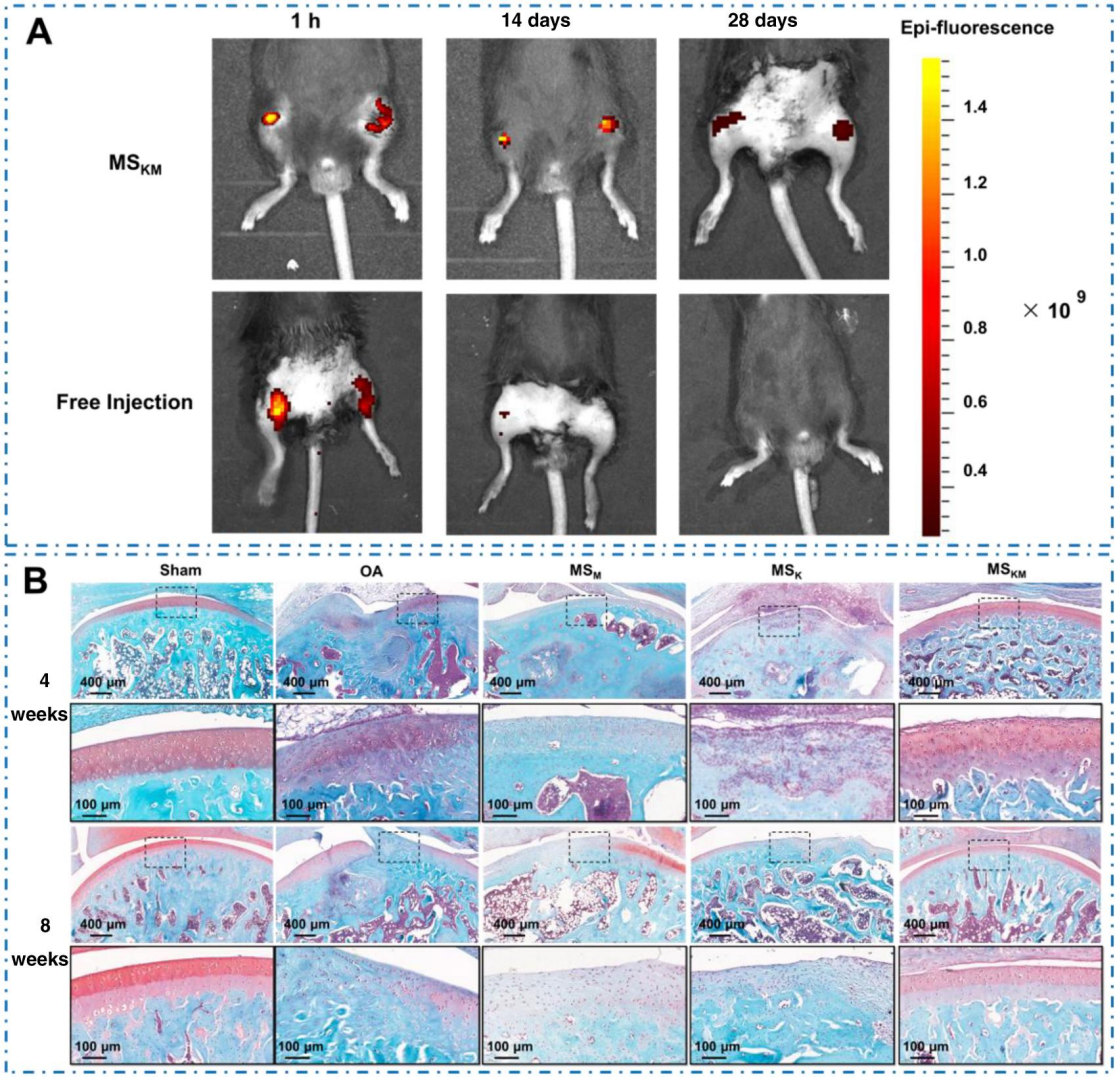
(**A**) *In vivo* retention of rhodamine B-labeled MS_KM_ and rhodamine B/KGN/PVA solution after 1 h, 14 days and 28 days of administration. (**B**) Safranin-O/fast green staining of the distal femora samples after different treatment with MS_K_, MS_M_, MS_KM_ for 4 and 8 weeks. Scale bars represent 400 and 100 μm. The images in the second and fourth rows represent the respective high-magnification images.

The 28-day retention patterns of MS_KM_ were also analyzed in this study, and the results demonstrated that the MS_KM_ group exhibited higher Rhodamine B-fluorescence intensity compared with that of the free injection group at all time points (Figure 7A). At 14 days of administration, the MPHS/PDLLA solution was undetectable in the free injection group, indicating a short retention period of the free MPHS/PDLLA solution in the injection site (Figure 7A). In contrast, the fluorescent signal from Rhodamine B-labeled MS_KM_ kept a 28-day period, demonstrating that the retention time of MPHS/PDLLA at the knee joint of mice was significantly longer than that of the injection group (Figure 7A). These findings suggested that MS_KM_ effectively slowed the drug-releasing speed and could serve as an effective controlled-releasing system for prolonged releasing of MPHS/PDLLA.

### Histological analysis

Histological analysis through Safranin O/Fast Green staining provided insights into the anti-OA performance of MS_KM_, MS_M_ and MS_K_ by evaluating GAGs expression and cartilage integrity at 4 and 8 weeks post-intra-articular injection. The samples in the OA group displayed typical OA pathology, such as surface irregularities, erosion of cartilage tissue, and GAGs loss (Figure 7B). Especially, extensive diffuse infiltration of inflammatory cells with markedly hyperchromatic nuclei was observed in the OA and MS_K_ groups (Figure 7B), indicating that the *in vivo* OA model was successfully created by MIA injection, and MS_K_ treatment had no positive influence on inflammation relief. After treatment with MS_KM_ and MS_M_, inflammatory cells were completely absent in the defect site. These results showed that controlled releasing of MPHS mitigated the inflammation caused by MIA.

The cartilage samples in the MS_M_ group remained intact and showed a similar amount of cartilage as the sham group. Nevertheless, a decrease in safranin O-positive tissues was noted in the MS_M_ group’s cartilage, indicating a reduction in GAGs in the MS_M_-treated samples (Figure 7B). In comparison, cartilage repair in the MS_K_ group was significantly improved, although the repair effect was still not optimistic. This is evidenced by the larger area of safranin O-positive tissues in the MS_K_-treated group when compared to the MS_M_ group, but still smaller than that in the sham group (Figure 7B). This suggests that controlled release of KGN alone may not sufficiently repair the damaged cartilage. The most promising results were observed in the MS_KM_ group, where the columnar structures of articular cartilage were well-preserved, and the surface integrity was maintained with no significant tissue deformation or exfoliation. Moreover, the samples in the MS_KM_ group demonstrated the highest GAGs expression among all the groups (Figure 7B), signifying effective cartilage matrix regeneration.

### Immunohistochemical staining

**Figure 8:**
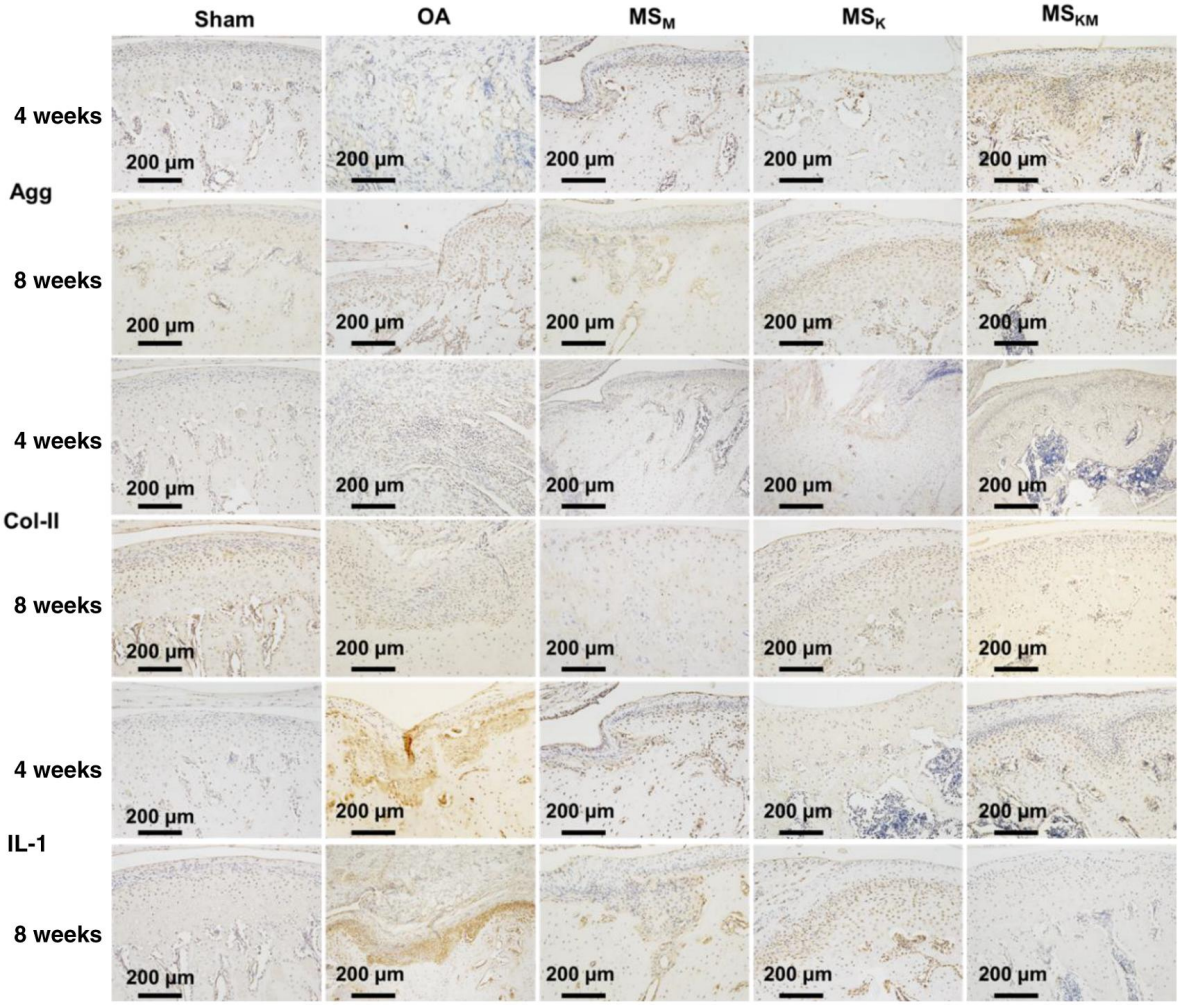
Immunohistochemical staining for agg, Col-II and IL-1 after 4 and 8 weeks of *in vivo* implantation. Scale bars represent 200 μm.

The sections of the distal femora were subjected to immunohistochemistry analysis for Agg, Col-II and IL-1 (Figure 8). The expression of Agg in the sham group is higher than that of the OA group (*P* < 0.05, Figure 8 and Figure S1A), suggesting that the amount of cartilage tissue suffering from OA is smaller than that of the normal tissues at 4 weeks of *in vivo* transplantation. After treatment with MS_M_ and MS_K_, however, the Agg expression was increased in the MS_M_ and MS_K_ groups (Figure 8 and Figure S1A). As to the MS_KM_ group, the Agg expression still accounted for the highest content among all other groups after 4 weeks of *in vivo* transplantation (*P* < 0.05, Figure 8 and Figure S1A), which basically conformed with the results of the Col-II expression. At 8 weeks, the Agg expression was slightly increased in OA, MS_M_ and MS_K_ groups when compared with those of the 4 weeks (*P* < 0.001, Figure 8 and Figure S1D) and almost equal with that of the MS_KM_ group. Those demonstrated that cartilage regeneration gradually recovered to a normal level after 8 weeks of healing, and MS_KM_ had preceded this process.

Compared with the OA and sham groups, the expression of Col-II was increased in the MS_K_ group after 4 weeks of *in vivo* transplantation (Figure 8 and Figure S1B). Rats treated with MS_KM_ displayed an excessive expression of Col-II-positive cartilage tissues at 4 weeks of *in vivo* transplantation (Figure 8 and Figure S1B), consistent with the results of Safranin O/fast green staining (Figure 7). After another 4 weeks of healing, the Col-II expression in the MS_KM_ group still accounted for the higher values than those of the OA, MS_M_ and MS_K_ groups at 8 weeks of *in vivo* transplantation (*P* < 0.05, Figure 8 and Figure S1E). Notably, Col-II expression in the samples of the MS_M_ group kept a relatively low content during the whole *in vivo* healing process (Figure 8 and Figure S1E), demonstrating that the sole releasing of MPHS is not enough to reverse the cartilage damage.

The expression of IL-1 in samples from the OA group was significantly higher than that of the sham group at 4 weeks of *in vivo* implantation (*P* < 0.01, Figure 8 and Figure S1C), demonstrating that the OA rat model was successfully created. After treatment with MS_M_, the IL-1 expression was reduced (Figure 8 and Figure S1C), confirming MPHS’s anti-inflammatory effects. However, IL-1 expression in the MS_KM_ group was significantly lower than that of the OA group (*P* < 0.01, Figure 8 and Figure S1C), illustrating the importance of the synergistic effect of KGN and MPHS in reducing OA-induced inflammation. However, in the MS_K_ group, IL-1 expression was slightly higher than in MS_KM_ although no significant statistical difference was found (*P* > 0.05, Figure 8 and Figure S1C). At 8 weeks of *in vivo* implantation, the IL-1 expression in the OA group still accounted for the highest content among all groups (Figure 8 and Figure S1F). Combined releasing of KGN and MPHS, however, not only restored the cartilage defects caused by OA but also reduced the expression of IL-1 to a relatively low level at 8 weeks (Figure 8 and Figure S1F). In general, the aforementioned results indicated that the KGN/MPHS-loaded MS_M_ has a prominent performance in reducing the inflammation and promoting cartilage regeneration and presents a new therapeutic method for the OA treatment [38].

### *In vivo* toxicity of microspheres

**Figure 9:**
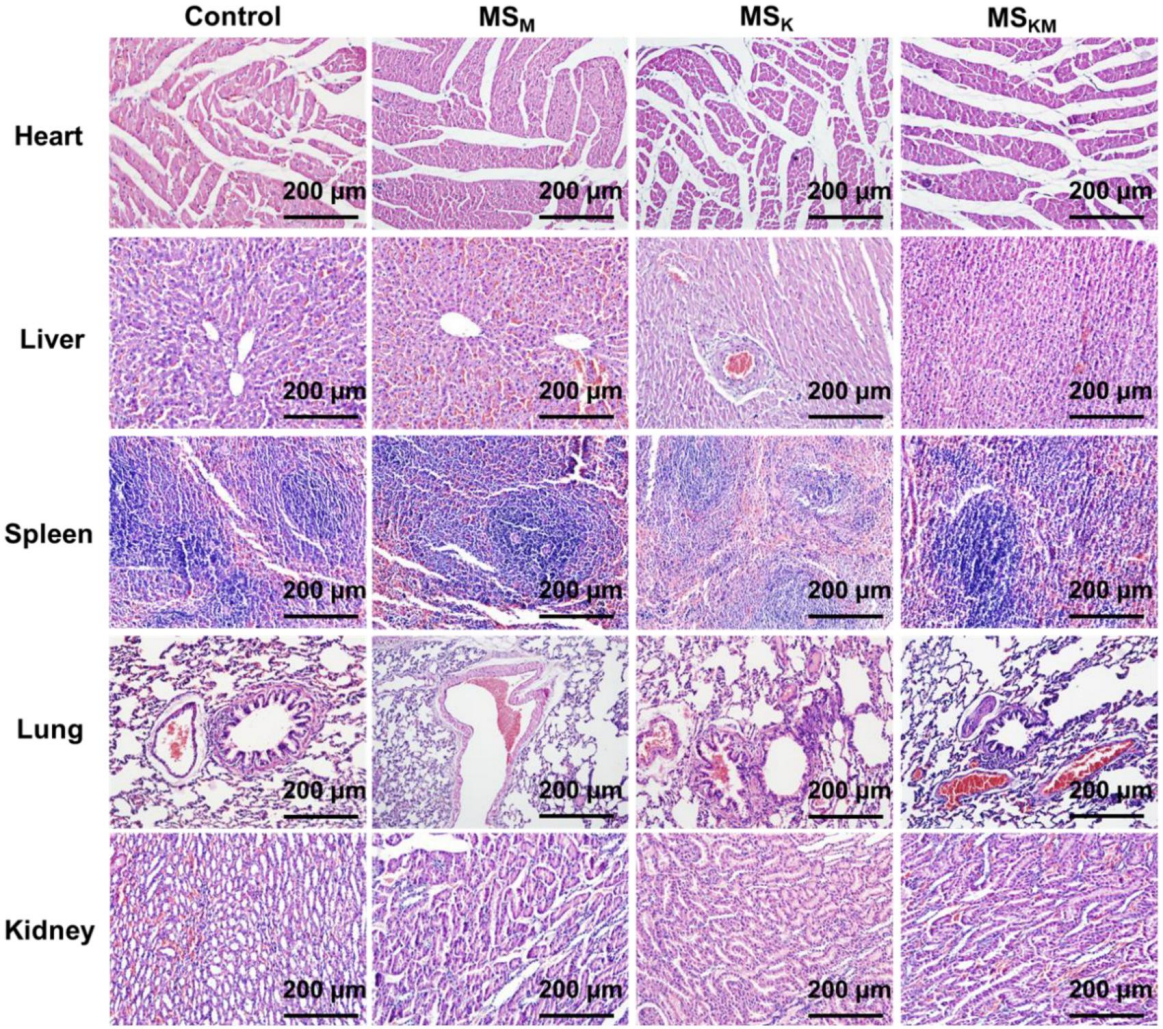
HE Staining of rat heart, liver, spleen, lung and kidney after 8 weeks of *in vivo* implantation. Scale bars represent 200 μm.

To evaluate the *in vivo* biosafety of MS_KM_, MS_M_ and MS_K_, HE staining was performed to analyze the toxic effects of microspheres on major organs (heart, liver, spleen, lung and kidney) of rats [39]. After 8 weeks of *in vivo* transplantation, there was no obvious inflammation, necrosis, or edema that could be observed in the heart, liver, spleen, lung, and kidney (Figure 9), indicating that the KGN-loaded, MPHS-loaded and KGN/MPHS-loaded microspheres pose no harm on the normal metabolism of animals.

## Conclusion

In this study, we developed PDLLA/PVA microspheres co-loaded with KGN and MPHS that enable sustained release of KGN and transient release of MPHS. *In vitro* experiments demonstrated that the coordinated release of KGN and MPHS effectively attenuated inflammation in IL-1β-treated BMSCs while promoting chondrogenic differentiation. Furthermore, intra-articular administration of the KGN/MPHS-loaded microspheres significantly reduced inflammatory responses and enhanced cartilage regeneration in a rat model of OA. Collectively, these findings highlight a versatile microsphere-based delivery platform for the dual release of KGN and MPHS and support its potential as a novel therapeutic strategy for OA.

## Supplementary Material

rbag027_Supplementary_Data

## References

[1] Wong BL , Bae WC, Gratz KR, Sah RL. Shear deformation kinematics during cartilage articulation: effect of lubrication, degeneration, and stress relaxation. Mol Cell Biomech 2008;5:197–206. 18751528 PMC2847289

[2] Gomoll AH , Minas T. The quality of healing: articular cartilage. Wound Repair Regen 2014;22 (Suppl. 1):30–8. 24813362 10.1111/wrr.12166

[3] Ma Z , Wu Y, Li G, Liu J, Geng Z, Su J. Extracellular vesicles-loaded DNA hydrogels: a promising candidate for cartilage organoids engineering. Chem Eng J 2023;477:147146.

[4] Xu X-L , Xue Y, Ding J-Y, Zhu Z-H, Wu X-C, Song Y-J, Cao Y-L, Tang L-G, Ding D-F, Xu J-G. Nanodevices for deep cartilage penetration. Acta Biomater 2022;154:23–48. 36243371 10.1016/j.actbio.2022.10.007

[5] Geng Z , Wang X, Yu Y, Ji L, Wang J, Liu C. Attenuating osteoarthritis by a high efficient anti-bone resorption injectable pH-responsive bisphosphonate-conjugated nano-apatite system. Chem Eng J 2021;420:127674.

[6] Jones IA , Togashi R, Wilson ML, Heckmann N, Vangsness CT Jr. Intra-articular treatment options for knee osteoarthritis. Nat Rev Rheumatol 2019;15:77–90. 30498258 10.1038/s41584-018-0123-4 PMC6390843

[7] Shen CY , Zhou QR, Wu X, Han XY, Zhang Q, Chen X, Lai YX, Bai L, Jing YY, Wang JH, Wang CL, Geng Z, Su JC. Accelerating cartilage regeneration with DNA-SF hydrogel sustained release system-based cartilage organoids. Mil Med Res 2025;12:39. 40722047 10.1186/s40779-025-00625-z PMC12302690

[8] Ge M , Zhu W, Mei J, Hu T, Yang C, Lin H, Shi J. Piezoelectric‐enhanced nanocatalysts trigger neutrophil N1 polarization against bacterial biofilm by disrupting redox homeostasis. Advanced Materials 2025;37:2409633. 10.1002/adma.202409633 39350533

[9] Song P , Cui Z, Hu L. Applications and prospects of intra-articular drug delivery system in arthritis therapeutics. J Control Release 2022;352:946–60. 36375618 10.1016/j.jconrel.2022.11.018

[10] Brown S , Kumar S, Sharma B. Intra-articular targeting of nanomaterials for the treatment of osteoarthritis. Acta Biomater 2019;93:239–57. 30862551 10.1016/j.actbio.2019.03.010 PMC6615949

[11] No YA , Seok J, Hyun MY, Kwon T-R, Oh CT, Choi EJ, Kim BJ. Long-term (24-month) safety evaluation of poly-DL-lactic acid filler injection for the nasolabial fold: a multicenter, open, randomized, evaluator-blind, active-controlled design. Plast Reconstr Surg 2015;135:1074e–5e. 10.1097/PRS.0000000000001247 25724056

[12] Zatorska M , Łazarski G, Maziarz U, Wilkosz N, Honda T, Yusa S-i, Bednar J, Jamróz D, Kepczynski M. Drug-loading capacity of polylactide-based micro-and nanoparticles–experimental and molecular modeling study. Int J Pharm 2020;591:120031. 33130219 10.1016/j.ijpharm.2020.120031

[13] Tang Q , Lim T, Shen L-Y, Zheng G, Wei X-J, Zhang C-Q, Zhu Z-Z. Well-dispersed platelet lysate entrapped nanoparticles incorporate with injectable PDLLA-PEG-PDLLA triblock for preferable cartilage engineering application. Biomaterials 2021;268:120605. 33360073 10.1016/j.biomaterials.2020.120605

[14] Zhou L , Gjvm VO, Malda J, Stoddart MJ, Lai Y, Richards RG, Ki‐wai Ho K, Qin L. Innovative tissue‐engineered strategies for osteochondral defect repair and regeneration: current progress and challenges. Adv Healthc Mater 2020;9:e2001008. 33103381 10.1002/adhm.202001008

[15] Johnson K , Zhu S, Tremblay MS, Payette JN, Wang J, Bouchez LC, Meeusen S, Althage A, Cho CY, Wu X, Schultz PG. A stem cell–based approach to cartilage repair. Science (1979) 2012;336:717–21. 10.1126/science.1215157 22491093

[16] Kang ML , Ko J-Y, Kim JE, Im G-I. Intra-articular delivery of kartogenin-conjugated chitosan nano/microparticles for cartilage regeneration. Biomaterials 2014;35:9984–94. 25241157 10.1016/j.biomaterials.2014.08.042

[17] Mohan G , Magnitsky S, Melkus G, Subburaj K, Kazakia G, Burghardt AJ, Dang A, Lane NE, Majumdar S. Kartogenin treatment prevented joint degeneration in a rodent model of osteoarthritis: a pilot study. J Orthop Res 2016;34:1780–9. 26895619 10.1002/jor.23197 PMC6348064

[18] Chen P , Liao X. Kartogenin delivery systems for biomedical therapeutics and regenerative medicine. Drug Deliv 2023;30:2254519. 37665332 10.1080/10717544.2023.2254519 PMC10478613

[19] Zare P , Pezeshki-Modaress M, Davachi SM, Zare P, Yazdian F, Simorgh S, Ghanbari H, Rashedi H, Bagher Z. Alginate sulfate-based hydrogel/nanofiber composite scaffold with controlled kartogenin delivery for tissue engineering. Carbohydr Polym 2021;266:118123. 34044939 10.1016/j.carbpol.2021.118123

[20] Gao J , Su Y, Wang Z. Remote Co-loading of amphipathic acid drugs in neutrophil nanovesicles infilled with cholesterol mitigates lung bacterial infection and inflammation. Biomaterials 2023;296:122071. 36878092 10.1016/j.biomaterials.2023.122071 PMC9973434

[21] Ding Y , Lv B, Zheng J, Lu C, Liu J, Lei Y, Yang M, Wang Y, Li Z, Yang Y, Gong W, Han J, Gao C. RBC-hitchhiking chitosan nanoparticles loading methylprednisolone for lung-targeting delivery. Journal of Controlled Release 2022;341:702–15. 34933051 10.1016/j.jconrel.2021.12.018 PMC8684098

[22] Conaghan PG , Cook AD, Hamilton JA, Tak PP. Therapeutic options for targeting inflammatory osteoarthritis pain. Nat Rev Rheumatol 2019;15:355–63. 31068673 10.1038/s41584-019-0221-y

[23] Ge M , Ding Y, Hu T, Chen Y, Shahin V, Li B, Huang T, Qian Y, Zhou Z, Tao Y, Xie R, Tan C, Lin H, Shi J. Nanomedicine-enabled next-generation therapeutics for spinal cord injury. Materials Today 2025;86:522–47.

[24] Cheng G , Ma X, Li J, Cheng Y, Cao Y, Wang Z, Shi X, Du Y, Deng H, Li Z. Incorporating platelet-rich plasma into coaxial electrospun nanofibers for bone tissue engineering. Int J Pharm 2018;547:656–66. 29886100 10.1016/j.ijpharm.2018.06.020

[25] Zhu Y , Jiang S, Xu D, Cheng G, Shi B. Resveratrol-loaded co-axial electrospun poly (ε-caprolactone)/chitosan/polyvinyl alcohol membranes for promotion of cells osteogenesis and bone regeneration. Int J Biol Macromol 2023;249:126085. 37536411 10.1016/j.ijbiomac.2023.126085

[26] Xue S , Zhou X, Sang W, Wang C, Lu H, Xu Y, Zhong Y, Zhu L, He C, Ma J. Cartilage-targeting peptide-modified dual-drug delivery nanoplatform with NIR laser response for osteoarthritis therapy. Bioact Mater 2021;6:2372–89. 33553822 10.1016/j.bioactmat.2021.01.017 PMC7844135

[27] Yang J , Zhu Y, Wang F, Deng L, Xu X, Cui W. Microfluidic liposomes-anchored microgels as extended delivery platform for treatment of osteoarthritis. Chem Eng J 2020;400:126004.

[28] Yamaura K , Sather NA, Metlushko A, Nishimura H, Pavlović RZ, Hambright S, Ravuri SK, Philippon MJ, Stupp SI, Bahney CS, Huard J. Sustained-release losartan from peptide nanofibers promotes chondrogenesis. Front Bioeng Biotechnol 2023;11:1122456. 36814717 10.3389/fbioe.2023.1122456 PMC9939695

[29] Chen T , Peng Y, Hu W, Shi H, Li P, Que Y, Qiu J, Qiu X, Gao B, Zhou H, Chen Y, Zhu Y, Li S, Liang A, Gao W, Huang D. Irisin enhances chondrogenic differentiation of human mesenchymal stem cells via Rap1/PI3K/AKT axis. Stem Cell Research & Therapy 2022;13:392. 35922833 10.1186/s13287-022-03092-8 PMC9351134

[30] Seo B-B , Kwon Y, Kim J, Hong KH, Kim S-E, Song H-R, Kim Y-M, Song S-C. Injectable polymeric nanoparticle hydrogel system for long-term anti-inflammatory effect to treat osteoarthritis. Bioact Mater 2022;7:14–25. 34466714 10.1016/j.bioactmat.2021.05.028 PMC8377411

[31] Li J , Liu N, Huang Z, Wang W, Hou D, Wang W. Intra-articular injection of loaded sPL sustained-release microspheres inhibits osteoarthritis and promotes cartilaginous repairs. J Orthop Surg Res 2021;16:646–9. 34717689 10.1186/s13018-021-02777-9 PMC8557014

[32] Sun Z , Gu X, Hao T, Liu J, Gao R, Li Y, Yu B, Xu H. Intra-articular injection PLGA blends sustained-release microspheres loaded with meloxicam: preparation, optimization, evaluation in vitro and in vivo. Drug Deliv 2022;29:3317–27. 36369759 10.1080/10717544.2022.2144545 PMC9665077

[33] Zhang Q , Dehaini D, Zhang Y, Zhou J, Chen X, Zhang L, Fang RH, Gao W, Zhang L. Neutrophil membrane-coated nanoparticles inhibit synovial inflammation and alleviate joint damage in inflammatory arthritis. Nat Nanotechnol 2018;13:1182–90. 30177807 10.1038/s41565-018-0254-4

[34] Song P , Zhou C, Fan H, Zhang B, Pei X, Fan Y, Jiang Q, Bao R, Yang Q, Dong Z, Zhang X. Novel 3D porous biocomposite scaffolds fabricated by fused deposition modeling and gas foaming combined technology. Composites Part B: Engineering 2018;152:151–9.

[35] Augustine R , Zahid AA, Hasan A, Wang M, Webster TJ. CTGF loaded electrospun dual porous core-shell membrane for diabetic wound healing. Int J Nanomedicine 2019;14:8573–88. 31802870 10.2147/IJN.S224047 PMC6827515

[36] Chen H , Sun T, Yan Y, Ji X, Sun Y, Zhao X, Qi J, Cui W, Deng L, Zhang H. Cartilage matrix-inspired biomimetic superlubricated nanospheres for treatment of osteoarthritis. Biomaterials 2020;242:119931. 32145507 10.1016/j.biomaterials.2020.119931

[37] Au TYK , Yip RKH, Wynn SL, Tan TY, Fu A, Geng YH, Szeto IYY, Niu B, Yip KY, Cheung MCH, Lovell-Badge R, Cheah KSE. Hypomorphic and dominant-negative impact of truncated SOX9 dysregulates hedgehog–wnt signaling, causing campomelia. Proceedings of the National Academy of Sciences 2023;120:e2208623119. 10.1073/pnas.2208623119 PMC9910594 36584300

[38] Yao Y , Wei G, Deng L, Cui W. Visualizable and lubricating hydrogel microspheres via NanoPOSS for cartilage regeneration. Advanced Science 2023;10:2207438. 36973540 10.1002/advs.202207438 PMC10214257

[39] Luo Y , Ruan Z, Guo Z, Chen Y, Lin H, Ge M, Zhu C. Electron orbital hybridization‐enhanced copper‐nanocatalysis for anti‐infection. Adv Funct Materials 2024;34:2313742.

