# Supplementary material for: Poly (d, l-lactide)/polyvinyl alcohol-based injectable microspheres with inflammation alleviation and cartilage regeneration enhancement for treatment of temporomandibular joints osteoarthritis
Source: Regen Biomater. 2026 Mar 2;13:rbag027. doi: 10.1093/rb/rbag027 (PMC13049186; doi:10.1093/rb/rbag027)
Supplement: rbag027_Supplementary_Data [file rbag027_supplementary_data.docx]

Table. S1

| *Target name* | *Direction* | *Primer sequence* |
| --- | --- | --- |
| *IL-6* | *Forward* | *CTTCCAGCCAGTTGCCTTCTTG* |
|  | *Reverse* | *TGGTCTGTTGTGGGTGGTATCC* |
| *TNF-α* | *Forward* | *CACGCTCTTCTGTCTACTGAACTTC* |
|  | *Reverse* | *GGGCTACGGGCTTGTCACTC* |
| *Sox 9* | *Forward* | *GGCGGAGGAAGTCGGTGAAG* |
|  | *Reverse* | *AGATGGCGTTAGGAGAGATGTGAG* |
| *Acan* | *Forward* | *CACAGGCAGCACAGACACTTC* |
|  | *Reverse* | *GGAGTCAAGGTCGCCAGAGG* |
| *Col-2a1* | *Forward* | *ACGCTCAAGTCGCTGAACAAC* |
|  | *Reverse* | *AATCCAGTAGTCTCCGCTCTTCC* |
| *GAPDH* | *Forward* | *AAGTTCAACGGCACAGTCAAGG* |
|  | *Reverse* | *GACATACTCAGCACCAGCATCAC* |


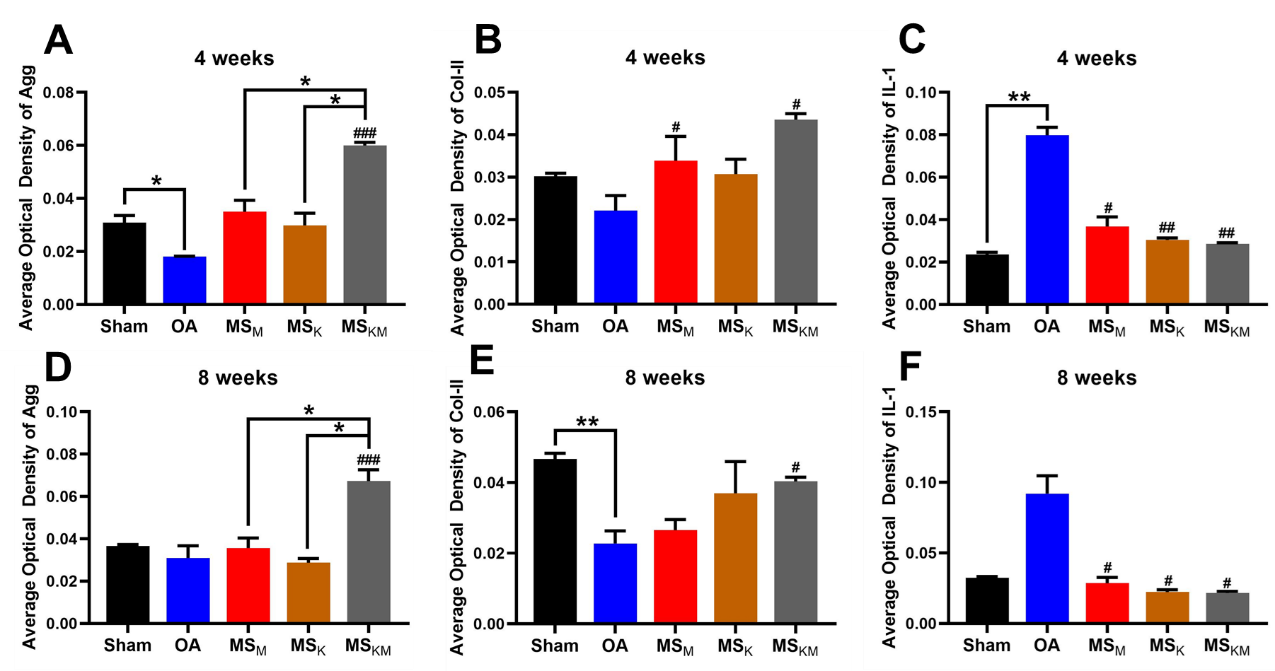


Fig. S1. Quantification of Agg (A, D), Col-II (B, E) and IL-1 (C, F) expression of sections in knee joints in the sham, OA and MS_K_, MS_M_, MS_KM_ groups after 4 and 8 weeks of *in vivo* implantation. Data are presented as mean ± SD. **P* < 0.05; ***P* < 0.01. *#*, *##* and *###* indicate *P <* 0.05, *P <* 0.01 and *P <* 0.001 when compared with the OA group, respectively.
